# Cabergoline therapy for Macroprolactinoma during pregnancy: A case report

**DOI:** 10.1186/1756-0500-5-606

**Published:** 2012-10-31

**Authors:** Hira Shahzad, Aisha Sheikh, Lumaan Sheikh

**Affiliations:** 1 Medical College, Aga Khan University Hospital, Karachi, Pakistan; 2 Department of Medicine, Aga Khan University Hospital, Karachi, Pakistan; 3 Section of Feto-maternal Medicine and Neonatal Health Division of Woman and Child Health, Aga Khan University Hospital, Karachi, Pakistan

## Abstract

**Background:**

We assessed the safety of Cabergoline therapy during pregnancy in a lady with hyperprolactinemia intolerant to Bromocriptine.

**Case presentation:**

We report the case of a 31 year old lady who presented to us with uncontrolled hyperprolactinemia. A pituitary Macroadenoma was demonstrated by MRI. Due to intolerance to Bromocriptine, Cabergoline was started. The patient improved and subsequently conceived. MRI in the second trimester demonstrated further reduction in the tumor size. It was decided to continue Cabergoline throughout pregnancy to ensure further reduction in tumor size until delivery and to hold Cabergoline during postpartum period to allow for an adequate interval of breastfeeding. At 37 weeks of gestation, the patient delivered a healthy baby.

**Conclusion:**

We were able to safely treat macroprolactinemia in our patient during pregnancy with cabergoline. This case report contributes to the relatively meager data available which advocates the safety of cabergoline therapy in pregnant hyperprolactinemic patients.

## Background

Prolactinomas are the most common hormone secreting pituitary adenomas and comprise 40% of all pituitary tumors [1,2]. Until the mid 1980s, surgery was the preferred treatment of choice in patients with macroprolactinomas [2]. With the introduction of Bromocriptine (BRC) in 1972 this trend changed [3]. Trials proved that BRC lowered prolactin levels efficiently, improved symptoms and helped in reduction of the size of tumor itself. Usually drugs are stopped once a patient becomes pregnant to limit fetal exposure. At this point in time, data of over 6000 pregnancies with BRC evaluated in this fashion is available [4]. Cabergoline (CAB) is another drug belonging to the class of dopamine agonists that was approved for use in 1985 which is usually preferred over BRC due to its higher effectiveness in prolactin suppression and tumor reduction [5]. It has been found to be effective in patients who are refractory to BRC [6]. Moreover, its longer half life requires less frequent dosage, and a more feasible side effect profile have resulted in increased compliance by patients [6]. However literature regarding the safety of CAB during pregnancy is lacking [4]. Therefore CAB is not regarded as the first line drug and is used only as an alternative when BRC therapy fails [6]. We are reporting this case in order to contribute to the relatively meager data available to advocate the safety of cabergoline therapy in pregnant patients with hyperprolactinemia.

## Case presentation

A 31 year old lady, mother of three children, presented to the endocrinology clinic with an eight year history of hyperprolactinemia. Her prolactin levels at that time were found to be high, and according to the patient the MRI was normal and showed no evidence of a pituitary tumor. However these reports were not available to us for verification. She admitted to having been non-compliant with bromocriptine (BRC) 2.5 mg twice a day as had been prescribed to her due to tolerance issues. It is unclear as to how closely her prolactin (PRL) levels had been monitored.

**Figure 1:**
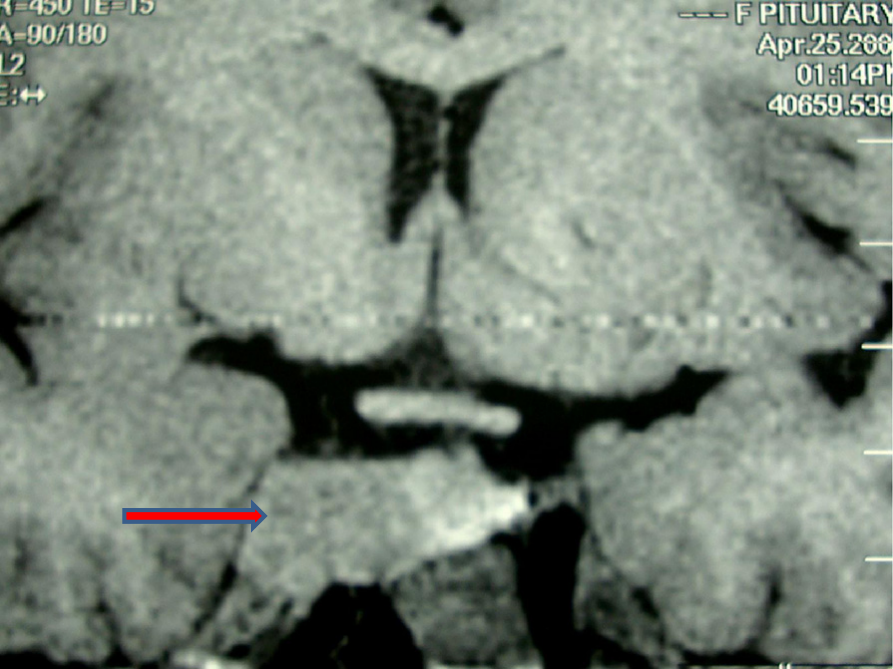
MRI (T1 weighted image) showing a Pituitary Macroadenoma measuring 2.2 cm × 2 cm × 1.3 cm (marked with a red arrow), with minimal supra-sellar extension, involving the right cavernous sinus with encasement of internal carotid artery and extending into the optic canal abutting the optic chiasm superiorly.

She had been taking BRC regularly for the last 3 months along with progesterone injections for withdrawal bleeding. PRL was 1300ng/dl (1.9 – 25 ng/ml). On physical examination, her body mass index (BMI) was 29 kg/m^2^. Visual fields were full by confrontation. Breast examination revealed expressible galactorrhea. There was evidence of acanthosis nigricans. Magnetic Resonance Imaging (MRI) was advised which showed a Pituitary Macroadenoma measuring 2.2 cm × 2 cm × 1.3 cm with minimal suprasellar extension, involving the right cavernous sinus with encasement of internal carotid artery and extending into the optic canal abutting the optic chiasm superiorly. (Figure 1) Due to cost issues with cabergoline (CAB), she was given another trial of BRC starting with a low dose with the intention of raising it up to 2.5 mg thrice daily within a month.

Since PRL levels remained high necessitating a dose build up of BRC and the patient still complained of intolerance to BRC, CAB was started at a low dose of 0.25 mg once a week. Due to persistently high PRL levels, CAB was increased to 0.5mg twice weekly. Any efforts to increase the dose further failed since the patient was unable to tolerate it. PRL dropped to 40ng/dl after eight months of CAB initiation. Her menstrual cycles returned to normal. A repeat MRI demonstrated the pituitary mass which had now decreased to 2.0 × 1.7 × 0.7 cm. Patient improved symptomatically as well as biochemically.

Twenty months after her initial presentation to us, the patient conceived. Because of the fact that she had macroprolactinoma, she was advised to continue CAB at 0.5mg once weekly. She was also given supplemental folic acid 5 mg once daily and Meclizine for morning sickness. A viable pregnancy was confirmed with ultrasound and patient was referred for antenatal care.

**Figure 2:**
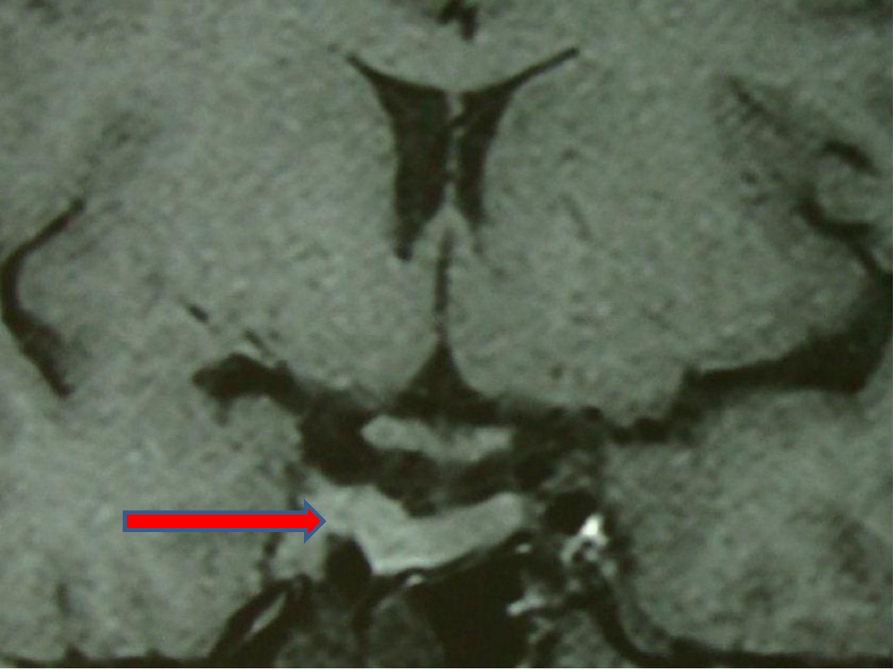
**Repeat MRI (T1 weighted image) in the second trimester during pregnancy was done in order to assess the tumor size.** It demonstrates considerable reduction in the tumor size, pituitary mass now being 1.8 × 1.0 cm (marked with a red arrow) as compared to the previous MRI shown in Figure 1.

Fetal Nuchal thickness ultrasound performed at 13 weeks to screen for major chromosomal abnormalities was normal. MRI was repeated in the second trimester in order to assess the tumor size. MRI without contrast demonstrated further reduction in the tumor size, pituitary mass now being 1.8 × 1.0 cm. (Figure 2) It was decided to continue CAB throughout pregnancy to ensure further reduction in tumor size until delivery and to hold CAB during postpartum period to allow for an adequate interval of breastfeeding. Perimetry was repeated every three months to check visual fields which remained normal. At 25 weeks of pregnancy, patient complained of headaches not effectively relieved with analgesics and therefore CAB was reduced to 0.25 mg weekly.

At 28 weeks of gestation, patient developed generalized pruritus and dark urine. Liver function tests (LFTs) showed raised levels of ALT (SGPT) = 281 (< 34 IU/L), AST (SGOT) = 220 (< 31 IU/L) and Alkaline Phosphatase= 211 (42–121). Total and direct bilirubin levels were within the normal range. Ultrasound of liver and gallbladder was normal. Hepatitis profile was also normal. Diagnosis of cholestatic jaundice was made (serum bile acids are not available in our laboratory). Patient received Urodeoxycholic acid 500 mg at thrice daily dosage. CAB was continued at 0.25 mg weekly throughout this time period.

At 37 + weeks of gestation labor was induced due to obstetric cholestasis. She had an assisted vaginal delivery due to abnormal fetal heart rate during the second stage of labor. A live baby boy weighing 3 kg was delivered with an Apgar score of 8 at 1 and 9 at 5 minutes. Her postpartum period was uneventful. Serum ALT levels checked after 10 days of delivery were normal. To allow breast feeding her Cabergoline was stopped. Her Prolactin level after 6 months of delivery while she was breast feeding her baby and was off cabergoline was 61.4 ng/ml. As she opted to continue breast feeding her baby further she was not advised to restart cabergoline. Her baby is doing well one year postpartum. The patient continues to remain amenorrheic since she delivered. Her follow up with a repeat Prolactin level is awaited.

## Discussion

BRC and CAB have been extensively compared in clinical trials and studies with respect to their side-effect profiles and the relative ease of achieving pregnancy in hyperprolactinemic women [4,7]. One such study compared BRC and CAB in two groups of patients with hyperprolactinemia and reported that the frequency of galactorrhea and irregular menstruation were found to be lower in women receiving CAB and pregnancy was more frequently achieved among these patients [7].

Once ovulation and fertility is reinstated in women with hyperprolactinemia, there are two main issues during the course of the pregnancy; firstly, the effects of dopamine agonists on early fetal development and the outcome of pregnancy, and secondly, the effect of hormonal milieu on the size of prolactinoma. Of the data available for BRC from 6000 pregnancies studied, it was found that there was no significant difference in the incidence of spontaneous abortions, ectopic pregnancies, trophoblastic diseases or multiple pregnancies and only 1.8% had congenital malformations [4,8,9]. CAB studies demonstrated similar results with a frequency of 2.2% congenital malformations [10] which is still lower than the incidence (3.0%) found in the general population [11].

Tumor growth during pregnancy is another concern in this population. Since a rise in estrogen levels induces prolactin synthesis and secretion, it subsequently leads on to lactotroph cell hyperplasia and an increase in tumor size. Moreover, stopping dopamine agonistic therapy during this time period in order to protect the fetus from adverse effects associated with its continuation, causes further growth in the size of prolactinoma. Keeping this in mind, we ordered a repeat MRI for our patient during the second trimester in order to decide whether to withhold CAB therapy during pregnancy. By continuing therapy, we could diminish the size of tumor further such that once she delivered the child, we could hold CAB while she breastfed.

In their study [10], Lebbe et al. evaluated 100 pregnancies for the risk of adverse outcomes in females previously diagnosed with hyperprolactinemia who were being treated with CAB for a month or more before conceiving. Even though all patients had been advised to discontinue CAB as soon as their pregnancies were confirmed, 13 continued with therapy for variable time period. Cumulative fetal dose was calculated as the product of dose of CAB at discontinuation and the length of gestation during which CAB was given. Spontaneous miscarriages occurred in 10% and three medical terminations of pregnancy were performed for fetal malformations (3%). The remaining 84 deliveries resulted in 88 infants, three of whom presenting with a malformation (3.4%). On comparison, a malformation rate of 6.3% was found in the control group. Postnatal development of the children was normal.

A study showed that 78% of the women who received CAB therapy before and/or during early pregnancy delivered, among which 97% were live infants and neonatal abnormalities were recorded in 9% of the cases [12]. The rates of spontaneous abortions, miscarriages, preterm deliveries, embryo-fetal anomalies and postnatal neurological deterioration in children born to hyperprolactinemic mothers receiving CAB have been found as not being significantly different from those in normal pregnancies [12,13].

M. Laloi-michelin et al. reported four cases of pregnant women who successfully received CAB therapy [6]. Two of these women had gastric intolerance with BRC and tolerated CAB well. A third patient was under treatment with Quinagolide and was switched to CAB due to development of abnormal visual fields. The last case was of a woman who developed persistent headache with BRC treatment and her MRI showed an increase in tumor size. She was switched to CAB in the eighth month of pregnancy following which her tumor regressed. It was suggested that CAB could be used as an alternative treatment only when BRC failed. Similarly, cases have been reported previously where CAB has been employed for treatment of prolactinomas in situations where BRC therapy has failed [14-16]. It has been deemed to be more effective and better tolerated than BRC and has been associated with the birth of normal full term infants.

We had initially switched our patient from BRC to CAB because of intolerance to the former, following which her Prolactin levels returned to normal limits and she conceived. Weighing the benefits of continuation of therapy during pregnancy against the risk of adverse fetal outcomes, it was decided to prolong CAB treatment throughout gestation at a lower dose. Literature review had shown that the evidence of fetal abnormalities and adverse pregnancy outcomes in such patients was similar to that in the normal population. Moreover, persistent therapy throughout pregnancy meant that the size of the tumor could be condensed to an extent which would later on enable us to withhold therapy after delivery to allow an innocuous period of six months for lactation. In addition, after delivery the effect of pregnancy on growth of macroprolactinoma was no longer a threat, the decision to stop CAB for lactation with a much reduced tumor size was taken. With the exception of cholestatic jaundice which occurred during the 28^th ^week of gestation, the pregnancy was uneventful. Our patient delivered a healthy baby at term.

## Conclusion

To conclude, CAB was used safely and effectively to treat macroprolactinoma during pregnancy in our case. However, it is important to discuss with patients about the limited data available on the use of CAB during pregnancy and associated potential adverse effects on the fetus.

## Consent

Written informed consent was obtained from the patient for publication of this case report and accompanying images. A copy of the written consent is available for review by the Editor-in-Chief of this journal.

## Abbreviations

BMI: Body mass index; BRC: Bromocriptine; CAB: Cabergoline; MRI: Magnetic Resonance Imaging; PRL: Prolactin.

## Competing interests

The authors declare that they have no competing interests.

## Authors’ contributions

HS analyzed and interpreted the patient data regarding cabergoline therapy in macroprolcatinemia. HS, AS and LS were all major contributors in writing the manuscript. All authors read and approved the final manuscript.

## References

[B1] Katznelson L Klibanski A Prolactinomas Cancer Treat Res 1997 89 41 55 10.1007/978-1-4615-6355-6_3 9204187

[B2] Colao A Pituitary tumours: the prolactinoma. Best practice & research Clin Endocrinol Metab 2009 2 575 596 10.1016/j.beem.2009.05.003 19945024

[B3] Besser GM Parke L Edwards CRW Forsyth IA MacNeilly AS Galactorrhea: successful treatment with reduction of plasma prolactin levels Br Med J 1972 3 669 672 10.1136/bmj.3.5828.669 4675488 PMC1786102

[B4] Molitch ME Prolactinomas and pregnancy Clin Endocrinol 2010 73 147 148 10.1111/j.1365-2265.2010.03823.x 20550542

[B5] Vance ML Evans WS Thorner MO Drugs five years later: bromocriptine Ann Int Med 1984 100 78 91 6229205 10.7326/0003-4819-100-1-78

[B6] Laloi-Michelin M Ciraru-Vigneron N Meas T Cabergoline treatment of pregnant women with macroprolactinomas Int J Gynaecol Obstet 2007 99 61 62 10.1016/j.ijgo.2007.04.027 17602689

[B7] Motazedian S Babakhani L Fereshtehnejad SM Mojthahedi K A comparison of bromocriptine & cabergoline on fertility outcome of hyperprolactinemic infertile women undergoing intrauterine insemination Indian J Med Res 2010 131 670 674 20516539

[B8] Krupp P Monka C Bromocriptine in pregnancy: safety aspects Klin Wochenschr 1987 65 823 827 10.1007/BF01727477 3657044

[B9] Krupp P Monka C Richter K The safety aspects of infertility treatments Program of the Second World Congress of Gynecology and Obstetrics 1988 Rio de Janeiro, Brazil

[B10] Lebbe M Hubinont C Bernard P Maiter D Outcome of 100 pregnancies initiated under treatment with cabergoline in hyperprolactinaemic women Clin Endocrinol 2010 73 236 242 10.1111/j.1365-2265.2010.03808.x 20455894

[B11] Canfield MA Honein MA Yuskiv N National estimates and race/ethnic-specific variation of selected birth defects in the United States, 1999–2001 Birth Defects Res A Clin Mol Teratol 2006 76 747 756 10.1002/bdra.20294 17051527

[B12] Colao A Abs R Bárcena DG Chanson P Paulus W Kleinberg DL Pregnancy outcomes following cabergoline treatment: extended results from a 12-year observational study Clin Endocrinol 2008 68 66 71 10.1111/j.1365-2265.2007.03000.x 17760883

[B13] Stalldecker G Mallea-Gil MS Guitelman M Alfieri A Ballarino MC Boero L Chervin A Danilowicz K Diez S Fainstein-Day P García-Basavilbaso N Glerean M Gollan V Katz D Loto MG Manavela M Rogozinski AS Servidio M Vitale NM Effects of cabergoline on pregnancy and embryo-fetal development: retrospective study on 103 pregnancies and a review of the literature Pituitary 2010 13 345 350 10.1007/s11102-010-0243-6 20676778

[B14] Bajwa SK Bajwa SJS Mohan P Singh A Management of prolactinoma with cabergoline treatment in a pregnant woman during her entire pregnancy Indian J Endocrinol Metab 2011 15 267 270 10.4103/2230-8210.84883 PMC3183510 22029039

[B15] Liu C Tyrrell JB Successful treatment of a large macroprolactinoma with cabergoline during pregnancy Pituitary 2001 4 3 179 185 10.1023/A:1015319007880 12138991

[B16] Forsbach-Sánchez G Tamez-Pérez HE Hernández-Herrera R Bafidis-Lechuga B Treatment of macroprolactinoma with cabergoline during pregnancy Rev Med Inst Mex Sequro Soc 2009 47 3 307 310 20141661

